# Harvesting Energy from the Counterbalancing (Weaving) Movement in Bicycle Riding

**DOI:** 10.3390/s120810248

**Published:** 2012-07-30

**Authors:** Yoonseok Yang, Jeongjin Yeo, Shashank Priya

**Affiliations:** 1 Biomedical Engineering, Chonbuk National University, Deokjin-dong Jeonju 664-14, Korea; E-Mail: ysyang@jbnu.ac.kr; 2 Healthcare Engineering, Chonbuk National University, Deokjin-dong Jeonju 664-14, Korea; E-Mail: yeojjin85@gmail.com; 3 Center for Energy Harvesting Materials and Systems (CEHMS), Virginia Tech, Blacksburg, VA 24061, USA

**Keywords:** energy harvesting, bicycle weaving, economy of human cycling

## Abstract

Bicycles are known to be rich source of kinetic energy, some of which is available for harvesting during speedy and balanced maneuvers by the user. A conventional dynamo attached to the rim can generate a large amount of output power at an expense of extra energy input from the user. However, when applying energy conversion technology to human powered equipments, it is important to minimize the increase in extra muscular activity and to maximize the efficiency of human movements. This study proposes a novel energy harvesting methodology that utilizes lateral oscillation of bicycle frame (weaving) caused by user weight shifting movements in order to increase the pedaling force in uphill riding or during quick speed-up. Based on the 3D motion analysis, we designed and implemented the prototype of an electro-dynamic energy harvester that can be mounted on the bicycle's handlebar to collect energy from the side-to-side movement. The harvester was found to generate substantial electric output power of 6.6 mW from normal road riding. It was able to generate power even during uphill riding which has never been shown with other approaches. Moreover, harvesting of energy from weaving motion seems to increase the economy of cycling by helping efficient usage of human power.

## 1. Introduction

A bicycle is rich source of kinetic energy and thus there have been continuous efforts to harvest electric energy from its movement [1]. A wheel-based dynamo has been implemented on many bicycles for powering the flashlights. Dynamos can generate large amounts of output power, however, the friction at the contact between a wheel and dynamo rotator significantly increases the mechanical work required for pedaling. Recently, a regenerative braking system for bicycle has been developed by researchers in MIT [2], which may provide a viable solution for portable electric power during bicycle riding without affecting the user performance, but the hub motor used as generator in the regenerative system adds extra weight and complexity to the bicycle itself, thereby limiting its usability. The most important factor to be considered in developing electric generator for bicycle is user convenience in terms of extra mechanical work and economy of cycling, which totally depends on human power [3]. This is why most of the recent energy harvesting researches applied on bicycle have been focusing on use of vibration harvesters instead of classic dynamos [4]. However, there has not been any successful experimental effort demonstrating the practically acceptable level of output electric power generated from bicycle in real environments. Most of the experiments so far have been conducted in the laboratory environment with periodic input signals which has limited applicability towards analyzing the user–harvester interaction [1]. Thus, a new approach towards harvesting the kinetic energy on bicycle platform accompanied by real field tests is not only desired but also essential for practical implementation.

In observing the riding movement of bicycle, we noticed that it makes a three-dimensional trajectory “like a weaving motion” rather than a simple planar movement [5]. This three-dimensional movement is natural to the bicycle ride that ordinary people, without mentioning professional riders, usually perform in order to use their power more economically by concentrating their body weight on each pedal each time during the stroke cycle while use the same movement at the same time to balance. This is more obvious during the uphill riding or while accelerating [6,7]. This weaving motion tilts the whole bicycle frame noticeably with the maximum motion produced at its handlebar which is the farthest spot from the ground. The energy contained in this handlebar motion is much greater than the vertical vibration component caused by the uneven road surface. Further, this natural side-to-side counter-balancing motion does not require extra pedaling but rather helps the economy of cycling by reducing labor in lower limb muscle [8]. Besides, the period of the weaving motion can be adjusted by riders so that the energy harvester can generate more electric power which is major advantage over other fixed frequency vibration sources.

This study aims to develop a novel energy harvesting concept that can generate electric power by using the weaving motion commonly performed in bicycle riding. Experimental results reported in this paper clearly demonstrate the feasibility of the concept.

## 2. Experimental Section

### 2.1. Measurement and Analysis of the Bicycle Weaving Motion

In order to understand the weaving motion, first it is necessary to obtain the dynamic information about the 3D movement by quantifying the measurement of a bicycle motion in real environment. This will provide the information necessary for correctly estimating the harvested output electrical power as well as the mechanical boundary conditions needed for proper design of an energy harvester with high electromechanical conversion efficiency. Unfortunately, there is not much data available in literature about the weaving motion which might be contained in the lateral components of bicycle frame motion. Therefore, we measured the acceleration of the bicycle weaving by using a MEMS-based 3-axis accelerometer and analyzed its amplitude and frequency characteristics.

**Figure 1.**
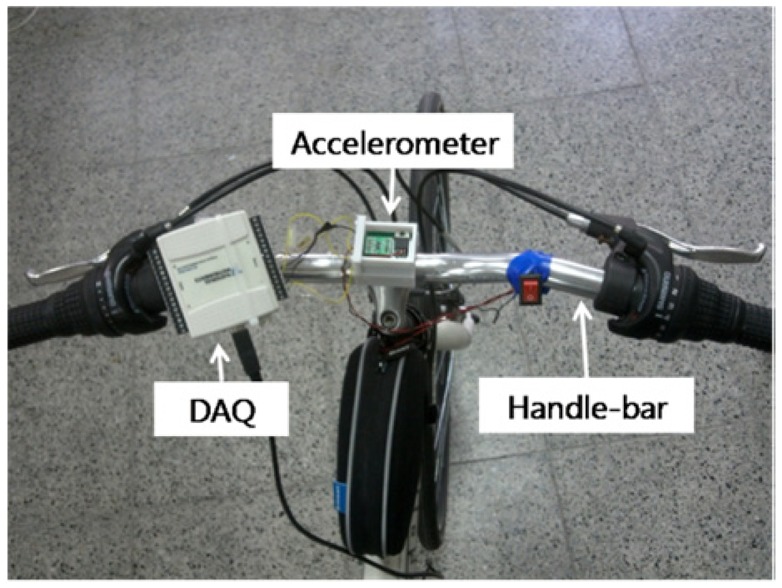
Accelerometer and data acquisition device installed on a bicycle handlebar for the weaving acceleration measurement.

**Figure 2.**
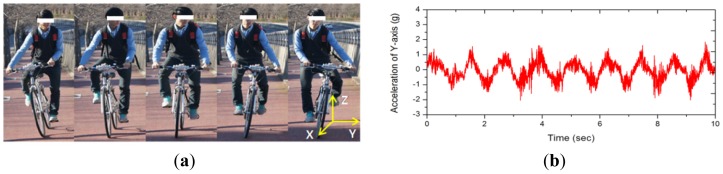
The weaving motion and its acceleration in experimental ride. (**a**) weaving motion accompanying uphill ride; (**b**) Y-axis acceleration

A city bike, *i.e.*, a bicycle designed for urban use, with weight of 14.5 kg and 27 inch wheel was used for measurement. Figure 1 shows the configuration of the 3-axis accelerometer, MMA7331LCT (freescale, Austin, TX USA) mounted at the top center of the handlebar and the data acquisition device, NI USB-6009 DAQ (National Instrument, Austin, TX, USA) to measure its acceleration. The measured data were stored in a laptop carried in the rider's backpack in real time through USB interface. A young (20 years old) male rider with height of 175 cm and weight of 62 kg participated in the experiment. The subject had normal bicycle riding skills. The driving road was gently sloped with inclination angle of about 10°. The participant was cycling up the slope at 10 km/h with almost periodic weaving motion as shown in Figure 2(a). Figure 2(b) displays a typical example of acceleration signals on the Y-axis of the accelerometer, which indicates the peak-to-peak acceleration of 3 g (1 g stands for earth gravity) and the center frequency of 1.2 Hz. Energy contained in such biomechanical motion can be effectively converted to electric energy by using a properly designed energy harvester [9].

### 2.2. Harvesting Energy from the Weaving Motion

Conventional energy harvesters are designed to generate the highest output electric power at the resonance frequency. However, in bicycle ride applications it is technically very difficult to fabricate an harvester having resonance frequency accurately matching that of source and human movements. Both of these are typically random, which implies that it is hard to operate the harvester at its resonance [10]. For this purpose, a nonlinear energy harvester has been developed which has the advantage of exhibiting relatively flat response over wide range of frequencies beyond its resonance and easier adjustment of the resonance condition by slight modification of its physical parameters [11–14]. It has potential to be used in efficient harvesting of low frequency biomechanical energy such as human motion and bicycle movement. Moreover, nonlinear response enables large peak output at some randomly triggering higher frequency input [11].

**Figure 3.**
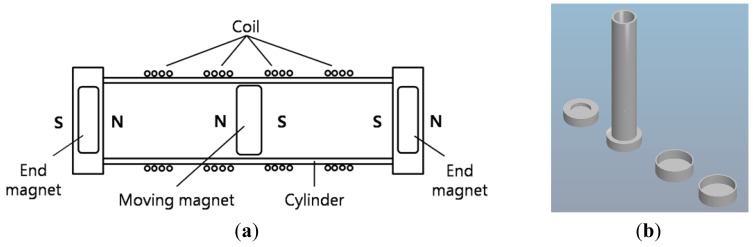
The nonlinear electromagnetic energy harvester (**a**) structure; (**b**) 3-D CAD model for rapid prototyping.

We designed and implemented a nonlinear electromagnetic harvester using neodymium-boron strong magnets as shown in Figure 3. The moving magnet in the center position in Figure 3(a) oscillates left-and-right between the two end magnets. Repulsive forces between the center and end magnets maintain the certain equilibrium separation. We found that a 10 mm thickness for the center magnet was adequate to prevent it from rotating within the cylindrical cavity. The diagonal length of 22.4 mm in the vertical cross-section of the moving magnet ensures proper positioning inside the cylinder with inner diameter of 20.2 mm. Marin *et al.* [15,16] have shown that the stiffness terms in such an system can be estimated by fitting the computational data with a 5th order non-linear curve and the damping constant is on the order of 0.0994. There were four solenoid windings on the outer surface of the cylindrical body for electromagnetic energy conversion. The length of the cylinder was selected to be suitable for being mounted on the bicycle handlebar without any interference with the steering. The physical dimension and design parameters are shown in Table 1. The power generated across the coil is given as [15]:

$$P={\left(\frac{Bl\dot{z}}{R_{L}+R_{e}}\right)}^{2}R_{L}$$

where the quantity *ż* represents the relative displacement of the center magnet with respect to coil, *R_L_* is the load resistance, *B* is the magnetic field, *l* is the length of coil, and *R_e_* is the coil resistance.

**Figure 4.**
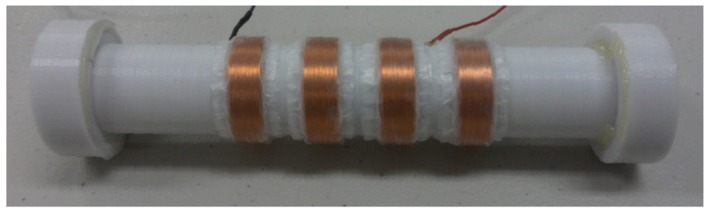
Developed nonlinear energy harvester.

Building upon the computational simulations available in literature on such nonlinear energy harvesters [15,16], we performed experiments to fine tune the parameters of the harvester for higher output by varying the dimensional and electrical variables as shown in Figure 3(b) and Figure 4. A rapid prototyping machine, FDM Vantage (Stratasys, Eden Prairie, MN, USA) was used for fabrication of the structural components. The displacement range of the center magnet has large effect on the generated electric power, however, it is difficult to be estimated under bicycle weaving motion because of the complex force vectors acting on the oscillating bicycle frame combined with gravity. This is why several prototypes were built to realize the optimum combination of the harvester variables. The gaps between the solenoid windings were found to be related to the thickness of moving magnet and its range of displacement. As a good approximation, the thickness of the center magnet is close to the separation between the coil windings.

**Figure 5.**
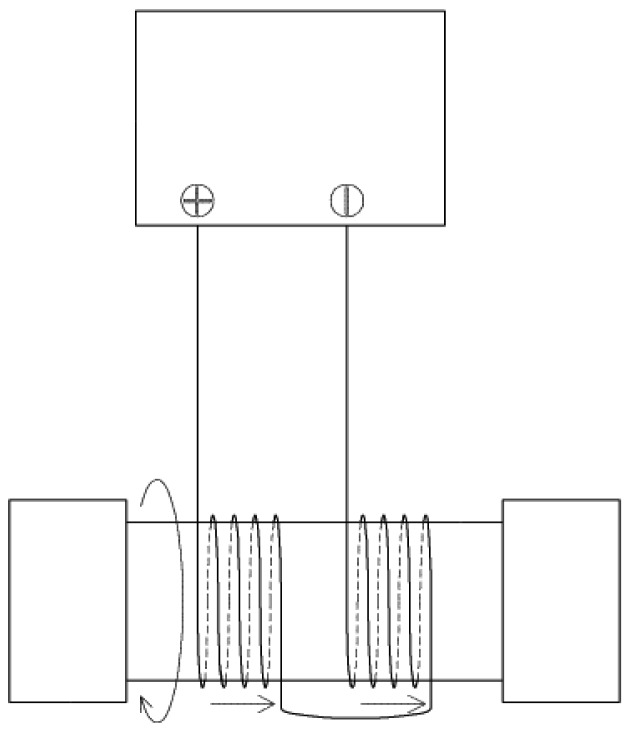
Phase-matching connection of neighboring solenoids by reversed series wiring.

The spatial gaps between the solenoids in Figure 3 and Figure 4 correspond to the size of the middle magnet in order to maintain the voltages induced by both edges of the magnet alive which, otherwise, would be canceling each other in the gapless winding [17]. Additionally, when the middle magnet is moving across the gap, the alternating current (AC) voltages induced in both neighboring solenoids have phases approximately opposite to each other. Therefore, two neighboring solenoids were connected in series but reversely wired for phase-matching as shown in Figure 5, which was helpful to increasing the voltage output by capturing difference between two opposite phased waveform. These results are consistent with that reported by Marin *et al.* [17] who have modeled this phenomenon in detail and validated it experimentally. Through FEM simulations it was shown that there are several regions within the single solenoid coil where no voltage is created due to cancellation of current transduction. The cancellation occurs because the direction of the magnetic field vectors at each end of the magnet is opposite. However, by splitting the single coil into three separate coils of similar thickness to that of the center magnet a significant increase in the transformation factor (*Bl*) can be obtained.

### 2.3. Experimental Verification

**Figure 6.**
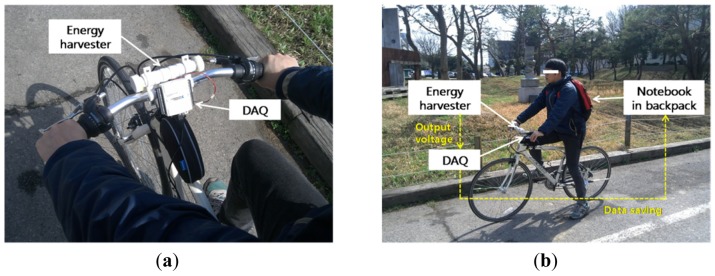
Experimental ride for the measurement of generated electricity.

**Figure 7.**
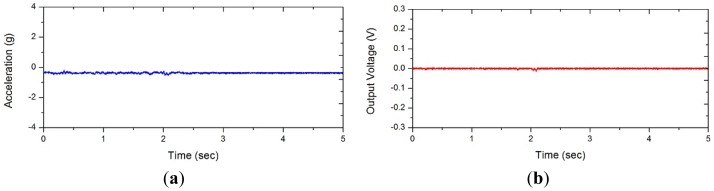
Signals from the control experiments. (**a**) Acceleration; (**b**) Harvester output signal.

The developed weaving harvester was mounted on the bicycle handlebar to measure the generated electric power from bicycling as shown in Figure 6. The output was connected to an 18 Ω external resistive load which approximately matched the internal resistance of the solenoid coils in the harvester. The voltage output was measured with the DAQ device and stored in the laptop computer carried in the rider's backpack. The accelerometer was also positioned under the harvester for simultaneous measurement of driving acceleration input. During these measurements, the bicycle rode the same slope as shown in Figure 2(a) that was used for acceleration measurement. Four young male participants were chosen without considering their cycling skills. We purposely designed some initial cycling period without the weaving motion, that is, control experiments in order to rule out any artifacts that may not belong to the weaving motion. Figure 7 displays one such typical result from the control experiments clearly demonstrating absence of any perturbation. This ensures that the harvested energy was not from noise or artifact but only that produced by the weaving motion.

**Figure 8.**
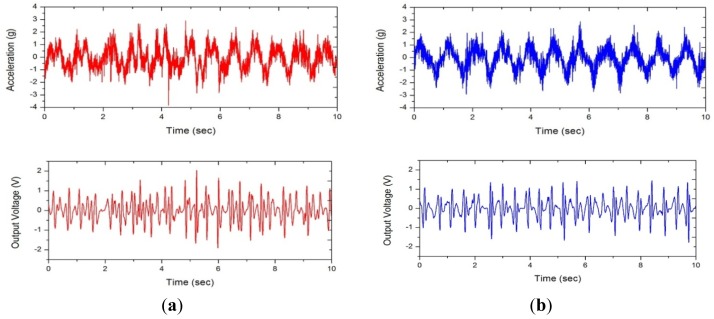

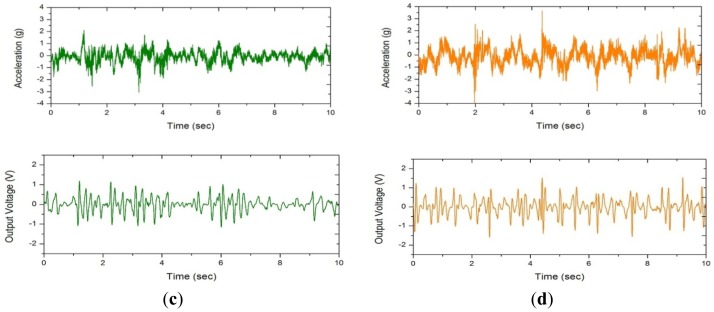
Acceleration and voltage waveform measured from each participant. (**a**) A; (**b**) B; (**c**) C; (**d**) D.

From the waveform in Figure 8, we can see that participants A and B performed relatively more consistent, *i.e.*, stabilized weaving motion than C and D during cycling. Consequently, the average electric power generated by the participants A and B is higher than that of C and D, as can be seen in Table 2. However, the generated electric power was no less than 6.6 mW, even when the rider did not keep bicycle in consistent weaving motion.

## 3. Results and Discussion

### 3.1. Frequency Characteristic of the Weaving Motion

**Figure 9.**
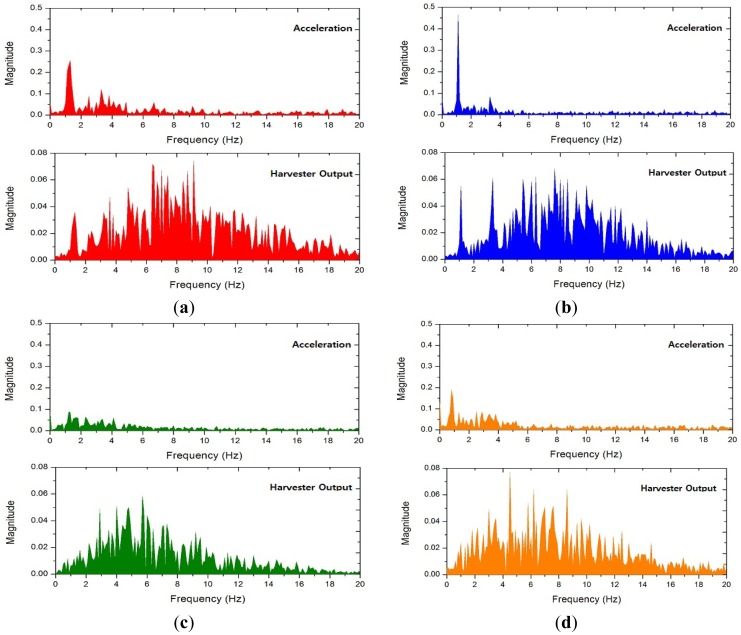
Power spectral density (PSD) estimation of acceleration and harvester output measured from each participant. (**a**) A; (**b**) B; (**c**) C; (**d**) D.

In general, low-frequency vibration is hard to convert into electric energy using an electromagnetic energy harvester since the rate of change in magnetic flux density is very low. However, the nonlinear harvester designed for the weaving motion showed a substantially large magnitude of electric power. This can be attributed to the harmonic frequency components contained in the weaving motion, which can be anticipated by its triangular-shaped waveform in Figure 8. Figure 9 shows the power spectral density (PSD) estimation of the waveforms shown in Figure 8, which confirms the existence and contribution of the harmonic components. For example, one can see many peaks over 0∼20 Hz frequency range in the acceleration plot of Figure 9(a) which reveals a fundamental frequency of about 1.2 Hz. Though they are small compared to the fundamental frequency component, there are other large peaks corresponding to higher frequencies in the harvester output plot of Figure 9(a) which greatly contribute towards the large output of the harvester. This proves the effectiveness of the weaving motion for harvesting energy and also its advantage over the other mechanisms proposed in literature [18]. Also, it is clear from Figure 9 that the nonlinear harvester has a larger response in the frequency region higher than the weaving fundamental frequency. This allows the nonlinear harvester to collect higher frequency components in the weaving motion as compared to that of a linear one. The linear harvester cuts-off higher frequency component beyond its resonance, and consequently, its output is smaller than that of the nonlinear one. Additionally, if the weaving motion gets faster, its harmonic frequencies as well as the fundamental frequency should also get higher. This implies that the nonlinear harvester is the best match for harvesting weaving motion since it shows gradually increasing response in higher frequency region as mentioned previously [11,19,20].

## 4. Conclusions

We have proposed a novel energy harvesting method that utilizes a bicycle's weaving motion for capturing kinetic energy. The feasibility of the concept was verified by experimental results which showed that more than 6.6 mW power can be generated under normal bicycling conditions. This energy harvesting approach can generate electric power even during the uphill riding without significant increase in mechanical work for user, which is a major differentiating factor from other approaches proposed in literature. The weaving motion is a counter-balancing response to the weight concentration on each one of the pedals for effective pedaling. Therefore, harvesting energy from weaving motion turns out to increase the economy of human cycling by helping efficient usage of human power [8,21]. Table 3 shows the average power consumption of typical portable electronic devices. Recent advances in ultra-low power electronics and smart power management technology has brought their power requirements below 15 mW [22]. This implies that the energy harvester developed in this study can supply the power for most of these portable electronic devices.

Ongoing studies are focused on improvements in the energy conversion efficiency of the electromagnetic harvester and finding additional energy source through dynamic analysis of the various movements in bicycle riding by using micro-inertial sensors. Further improvements will enable the powering of smart-phone and location-based service applications while bicycle riding.

## Figures and Tables

**Table 1.** Physical information of the participants.

**Parameter**	**Dimension**
Cylinder length (mm)	140
Cylinder inner diameter (mm)	20.2
Cylinder outer diameter (mm)	24.5
Moving magnet thickness (mm)	10
Moving magnet diameter (mm)	20
Moving magnet surface field (Gauss)	4770
End magnets thickness (mm)	5
End magnets diameter (mm)	10
End magnets surface field (Gauss)	4240
Coil thickness (mm)	0.3
Coil turns	200 (in each solenoid)
Coil layers	7 (in each solenoid)

**Table 2.** Parameters of the energy harvester.

**Participant**	**Age**	**Height (cm)**	**Weight (kg)**	**Cycling skills**	**Generated Power (mW)**
A	20	175	62	well	15.5
B	20	179	61	well	13.2
C	22	170	51	normal	6.6
D	24	171	49	normal	10.6

**Table 3.** Average power consumption of typical electronic devices and sensors.

**Electronics**	**Required Power**
GPS receiver chip	15 mW
Cell phone (standby)	8.1 mW
PPG sensor	1.47 mW
Humidity	1 mW
Pressure	0.5 mW
3D accelerometer	0.32 mW
Temperature	27 μW
A/D conversion	1 μW
RF transmission	sub μW
